# Fungal Keratitis: A Cross-Sectional Study of Corneal Ulcer Cases in a Tertiary Care Hospital in India

**DOI:** 10.7759/cureus.112406

**Published:** 2026-07-10

**Authors:** Samiksha Ghatage, Geeta Karande

**Affiliations:** 1 Medical Microbiology, Krishna Vishwa Vidyapeeth (Deemed to be University), Karad, IND; 2 Microbiology, Krishna Vishwa Vidyapeeth (Deemed to be University), Karad, IND

**Keywords:** corneal scrapings, corneal ulcer, fungal, keratitis, koh

## Abstract

Background and aim

Keratitis is an inflammatory condition of the cornea commonly caused by microbial infections and is an important cause of visual impairment worldwide. Fungal keratitis is an important cause of corneal infection and visual morbidity. This study was conducted to determine the fungal profile of corneal ulcers and to identify the predisposing factors associated with fungal keratitis.

Materials and methods

A cross-sectional study was conducted from August 2022 to August 2024 at a tertiary care center in Karad, Maharashtra, India. Patients presenting to the Ophthalmology Outpatient Department with corneal ulcers underwent detailed clinical evaluation and slit-lamp examination. Corneal scrapings were collected aseptically using a sterile surgical blade or needle and inoculated onto appropriate culture media for fungal isolation and identification. Data were entered into Microsoft Excel and analyzed using descriptive statistics. Results are expressed as frequencies and percentages.

Results

A total of 56 corneal ulcer samples were processed, of which eight (14.3%) yielded fungal isolates. The fungal pathogens identified included *Aspergillus fumigatus*, *Aspergillus flavus*, *Aspergillus niger*, *Mucor spp.*, and *Fusarium spp.* *Aspergillus fumigatus* was the most frequently isolated species.

Conclusion

Fungal keratitis was identified among corneal ulcer cases in this study. *A. fumigatus* was the most common isolate. Most culture-positive cases were observed in male patients with a farming background and a history of ocular trauma, particularly vegetative injury. Although limited by the small number of culture-positive cases and the absence of a comparator group, these findings emphasize the importance of early microbiological diagnosis and preventive measures in high-risk populations.

## Introduction

A wide range of microbial pathogens are responsible for corneal infections [1]. Eyes are vital sensory organs and are naturally protected against microbial contamination by intrinsic defense mechanisms. However, any disruption of these protective barriers can increase the risk of ocular diseases, potentially leading to significant visual impairment [2].

Microbial keratitis is a serious corneal infection caused by a wide range of microbial agents and should be considered a medical emergency [3]. Clinically, it manifests with symptoms such as pain, photophobia, redness, corneal infiltration, edema, ulceration, and anterior chamber inflammation. If not promptly treated, the condition may progress to endophthalmitis, corneal perforation, and even blindness [4].

According to the World Health Organization (WHO), microbial keratitis is a significant cause of corneal blindness and is often referred to as a “silent epidemic” worldwide [5]. The causative organism varies depending on the nature of the injury, environmental factors, and the individual’s sociodemographic background. Early and appropriate antimicrobial therapy is essential to prevent disease progression and complications [1].

Corneal blindness accounts for approximately 5.1% of global blindness, making it a leading cause after cataract, glaucoma, and age-related macular degeneration (AMD) [5]. Ocular trauma and corneal ulceration are among the major etiological factors contributing to corneal blindness. In many developing countries, including India, the use of traditional eye medicines remains a considerable public health concern and may exacerbate corneal infections. These non-sterile preparations can become contaminated, resulting in delayed recovery and facilitating the proliferation and dissemination of pathogenic microorganisms [6].

The etiology and epidemiology of corneal ulceration vary according to individual factors, corneal health, geographic location, and climatic conditions, and may also change over time. Therefore, an understanding of region-specific epidemiological characteristics, risk factors, and causative pathogens is essential for early diagnosis, prompt treatment, optimal disease management, and effective prevention strategies [7].

Despite improvements in literacy rates and economic status over the past few decades, microbial keratitis continues to affect a significant proportion of the population. Additionally, the widespread practice of self-medication among certain demographic groups remains an important contributing factor. Considering these aspects, this study was undertaken to determine the fungal profile of corneal ulcers and to identify the associated predisposing factors [8].

## Materials and methods

Study design and sample size

This observational cross-sectional study was conducted in the Department of Microbiology, Krishna Institute of Medical Sciences, Krishna Hospital and Medical Research Centre, Karad, Maharashtra, India, over a period of two years from August 2022 to August 2024. Although the study period started from August 2022 to August 2024, the research work was conducted only after obtaining Institutional Ethics Committee (IRB/IEC) approval dated 15/03/2023, which includes specimen collection and laboratory diagnostic work. Ethical approval for the study was obtained from the Institutional Ethics Committee of Krishna Institute of Medical Sciences (Deemed to be University), Karad (approval no. KIMSDU/IEC/02/2023; protocol no. 432/2022-2023). A total of 56 clinically suspected cases of corneal ulcer were included in the study.

A total of 56 patients presenting with corneal ulceration and clinical features suggestive of fungal keratitis were enrolled in the study. Patients of either sex were included. Cases with clinical findings suggestive of bacterial, viral, or parasitic keratitis were excluded. To avoid duplication of data, repeated isolates obtained from the same patient and specimen were excluded from analysis.

The study was conducted on patients admitted to the inpatient department/outpatient department (IPD/OPD) with any suspicion of clinical infection at Krishna Hospital and Medical Research Centre, Karad. Data were acquired from patients included in the study using a structured proforma. Details such as name, age, sex, address, IPD/OPD number, date of admission, clinical data including personal history, high-risk factors (farmers, construction workers, alcohol consumption, secondary infections), immunocompromised status, and details of clinical diagnosis were collected. A corneal ulcer was characterized as the presence of a corneal stromal infiltrate accompanied by an overlying epithelial disruption. Corneal specimens were systematically procured from patients presenting with ulcerative keratitis by an ophthalmologist under slit-lamp illumination using a sterile No. 15 surgical blade. Scrapings were collected from the edge of the ulcer before the initiation of any antimicrobial therapy, following the instillation of topical 4% xylocaine for local anesthesia [9].

**Figure 1:**
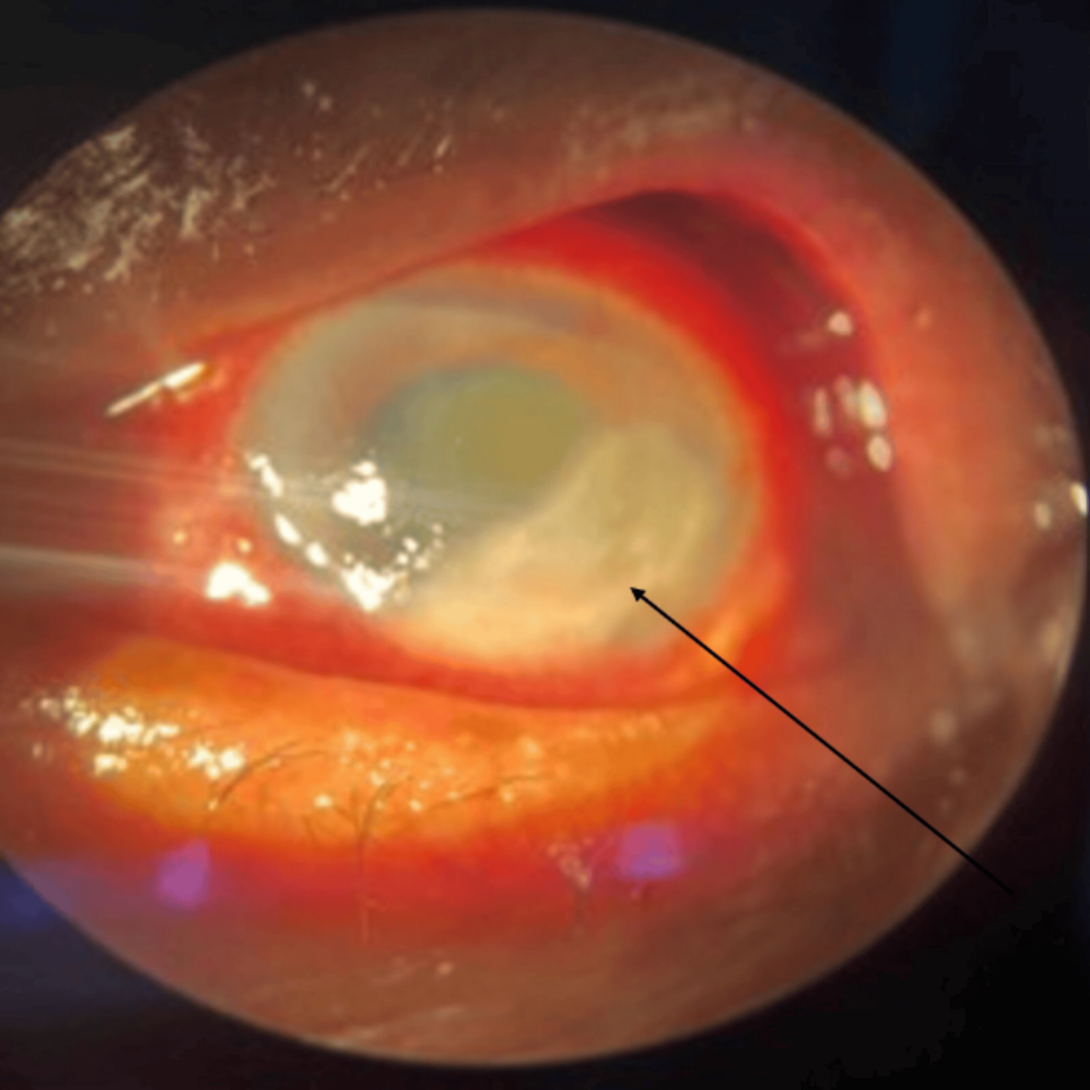
Corneal ulcer (arrow).

Figure 1 shows a corneal ulcer caused by fungal infection.

The material was obtained from the leading edge of an active ulcer and also from the base of the ulcer, smeared onto glass slides for potassium hydroxide (KOH) preparation, and directly inoculated onto Sabouraud dextrose agar (SDA) supplemented with chloramphenicol [9].

**Figure 2:**
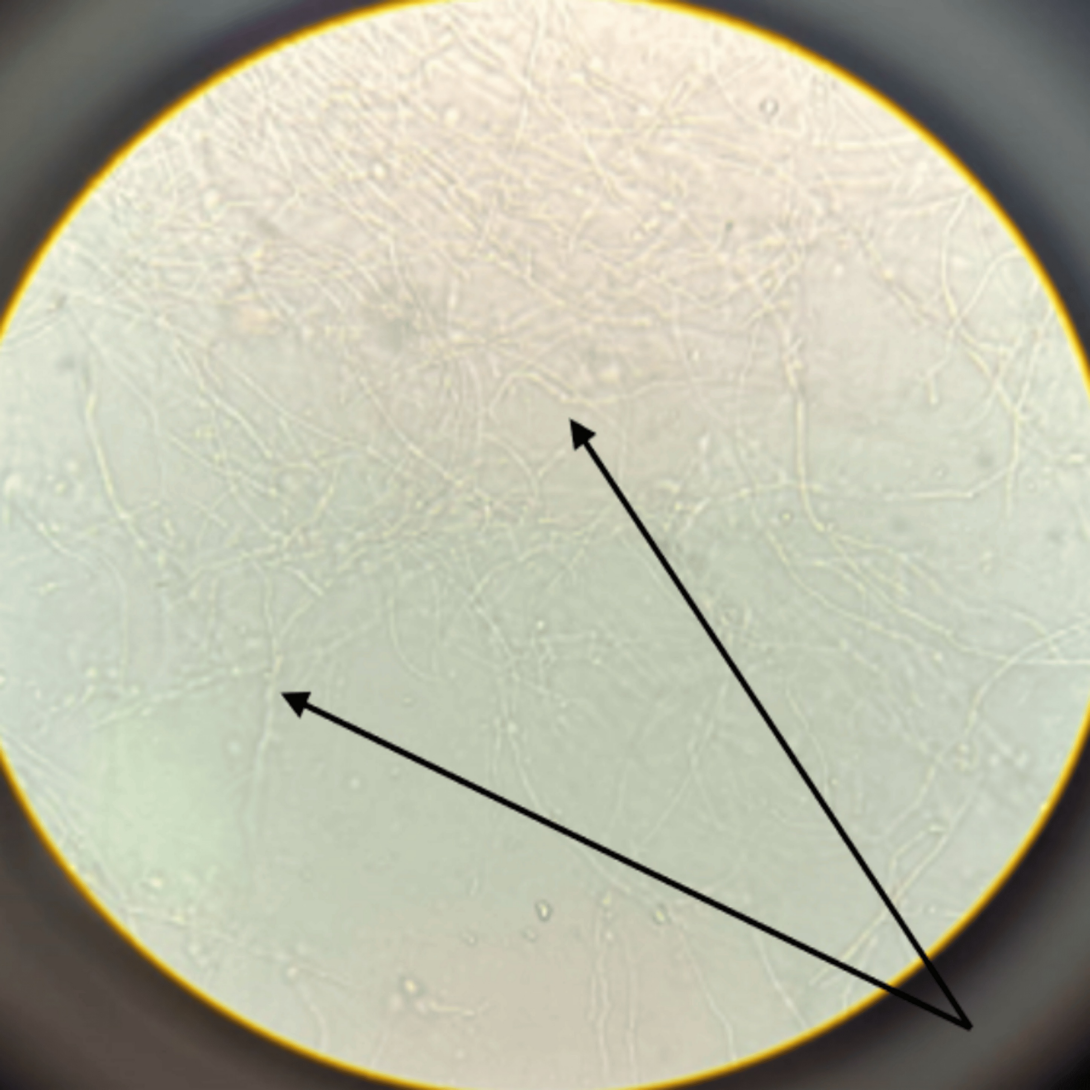
KOH mount showing fungal hyphae (arrows) using a 10× objective lens (100× total magnification). KOH: potassium hydroxide

Figure 2 shows the direct microscopic demonstration of fungal elements using a KOH mount.

The inoculated SDA plates were incubated at 25°C for three weeks and examined daily for evidence of fungal growth [9]. A fungal culture was considered positive when fungal growth was recovered from the corneal scraping specimen and identified by standard mycological methods based on colony morphology and lactophenol cotton blue (LPCB) mount findings. Strict aseptic precautions were followed during specimen collection and laboratory processing to minimize contamination. Colony morphology was recorded, and LPCB mounts were prepared for microscopic examination. Fungal isolates were identified based on their macroscopic characteristics on culture and microscopic morphology on LPCB mounts using standard mycological identification procedures described in the literature [10].

**Figure 3:**
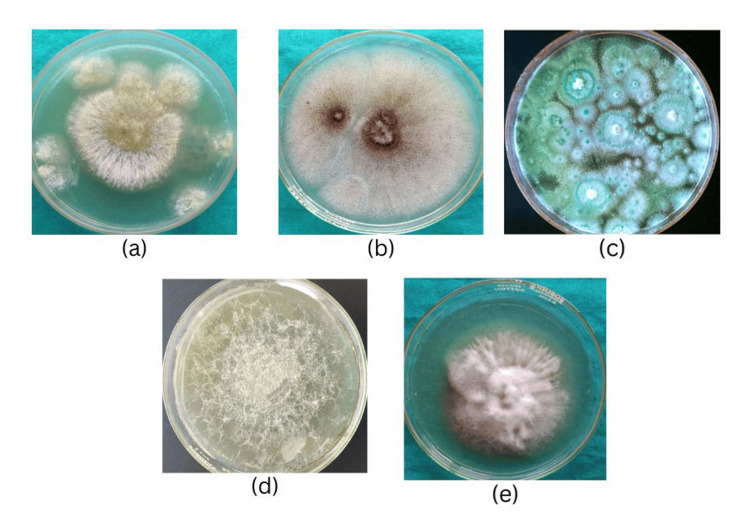
Macroscopic appearance of fungal isolates the SDA culture medium. (a) *Aspergillus flavus* (b) *Aspergillus niger* (c) *Aspergillus fumigatus* (d) *Mucor* spp. (e) *Fusarium* spp. SDA: Sabouraud dextrose agar

Figures 3a-3e show the isolated fungal elements grown on the SDA culture medium.

**Figure 4:**
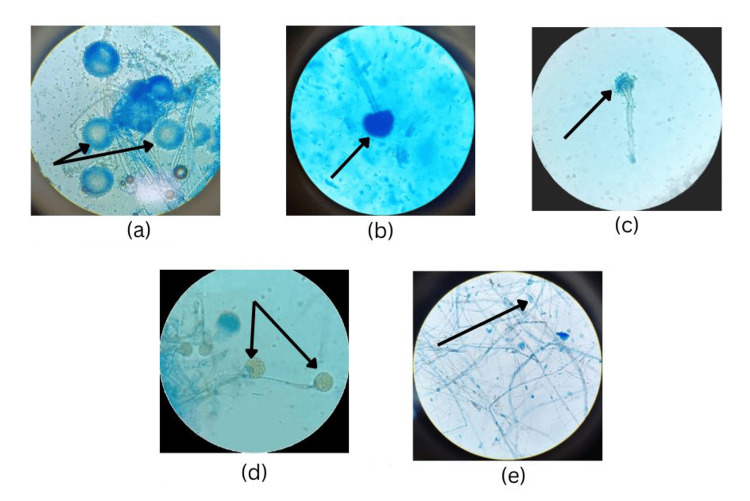
Lactophenol cotton blue (LPCB) mount showing microscopic appearance of fungal isolates (arrows) using a 40× objective lens (400× total magnification). (a) LPCB mount of *Aspergillus flavus*; arrows show conidia. (b) LPCB mount of *Aspergillus niger*; arrow shows a black conidium. (c) LPCB mount of* Aspergillus fumigatus*; arrow shows a hyaline conidium arising from the vesicle. (d) LPCB mount of *Mucor* spp.; arrows show sporangia. (e) LPCB mount of *Fusarium* spp.; arrow shows a macroconidium.

Figures 4a-4e show the microscopic morphology of fungal isolates on LPCB mounts from cases of fungal keratitis.

Statistical analysis

Data were entered into Microsoft Excel (Microsoft; Redmond, Washington, USA) and analyzed using descriptive statistics. Categorical variables are expressed as frequencies and percentages. The findings are presented in tables and figures wherever appropriate.

## Results

Over a two-year period (August 2022-August 2024), corneal scrapings from patients with corneal ulcers were collected in the Department of Ophthalmology and processed in the Department of Microbiology at Krishna Institute of Medical Sciences (Deemed to be University), Karad.

A total of 56 patients with corneal ulceration were included in the study. Males constituted 38/56 (67.9%) cases, while females accounted for 18/56 (32.1%) cases. The majority of patients belonged to the 41-60 years age group (23/56, 41.1%), followed by the 61-80 years age group (18/56, 32.1%). Trauma of unknown origin was the most frequently reported predisposing factor, accounting for 31/56 (55.4%) cases, followed by conjunctivitis (13/56, 23.2%) and leaf-cut injury (5/56, 8.9%). Of the 56 corneal ulcer cases evaluated, 8 (14.3%) were culture-positive for fungal keratitis, whereas 48 (85.7%) were fungal culture-negative.

Table 1 illustrates that 8 out of 56 samples (14.3%) were culture-positive for fungal keratitis. Of the eight culture-confirmed cases, five (62.5%) were also positive on direct microscopy using 10% KOH mount, indicating concordance between microscopy and culture findings. Males accounted for 5/8 (62.5%) of the culture-confirmed cases, while females accounted for 3/8 (37.5%).

**Table 1:** Gender-wise distribution of fungal keratitis Data are presented as n, number; (%), percentage

Gender	Number	Gender distribution
Male	5	62.5%
Female	3	37.5%
Total	8	100%

Table 2 presents the age-wise distribution of culture-confirmed fungal keratitis cases. The majority of culture-confirmed fungal keratitis cases were observed in the 41-60 years and 61-80 years age groups, accounting for 3/8 (37.5%) cases each, followed by the 81-100 years age group with 2/8 (25%) cases. No cases were observed in the 0-20 years and 21-40 years age groups.

**Table 2:** Age-wise distribution of fungal keratitis Data are presented as n, number; (%), percentage.

Age	Number	Age distribution
0-20	0	0%
21-40	0	0%
41-60	3	37.5%
61-80	3	37.5%
81-100	2	25%
Total	8	100%

Table 3 shows the occupation-wise distribution of fungal keratitis cases. Farmers constituted the majority of cases, accounting for 5/8 (62.5%), while workers accounted for 3/8 (37.5%) cases. This finding may reflect the predominantly rural and agricultural background of the study population.

**Table 3:** Occupation-wise distribution of fungal keratitis Data are presented as n, number; (%), percentage

Occupations	No. of patients	Percentage
Farmer	5	62.5
Worker	3	37.5
Total	8	100

**Figure 5:**
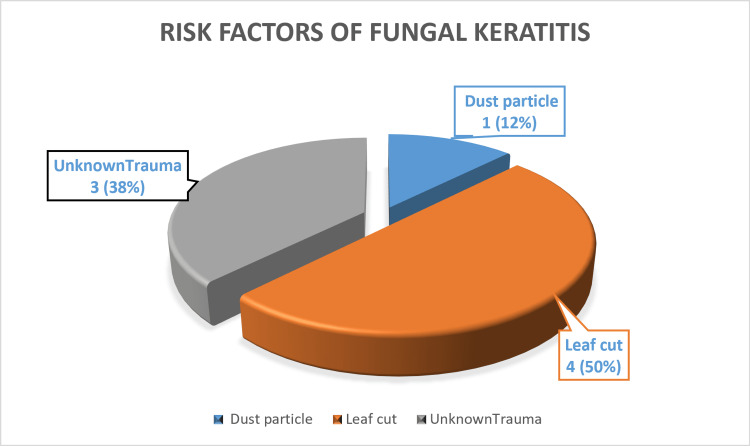
Risk factors for fungal keratitis. Data are presented as n, number; (%), percentage. No artificial intelligence (AI) tool was used to create the figure.

Figure 5 shows the distribution of predisposing factors among patients with fungal keratitis. Leaf-cut injury was the most common risk factor, accounting for 4/8 (50%) cases, followed by trauma of unknown origin in 3/8 (38%) cases. Dust particle injury was the least frequently reported risk factor, observed in 1/8 (12%) case.

**Figure 6:**
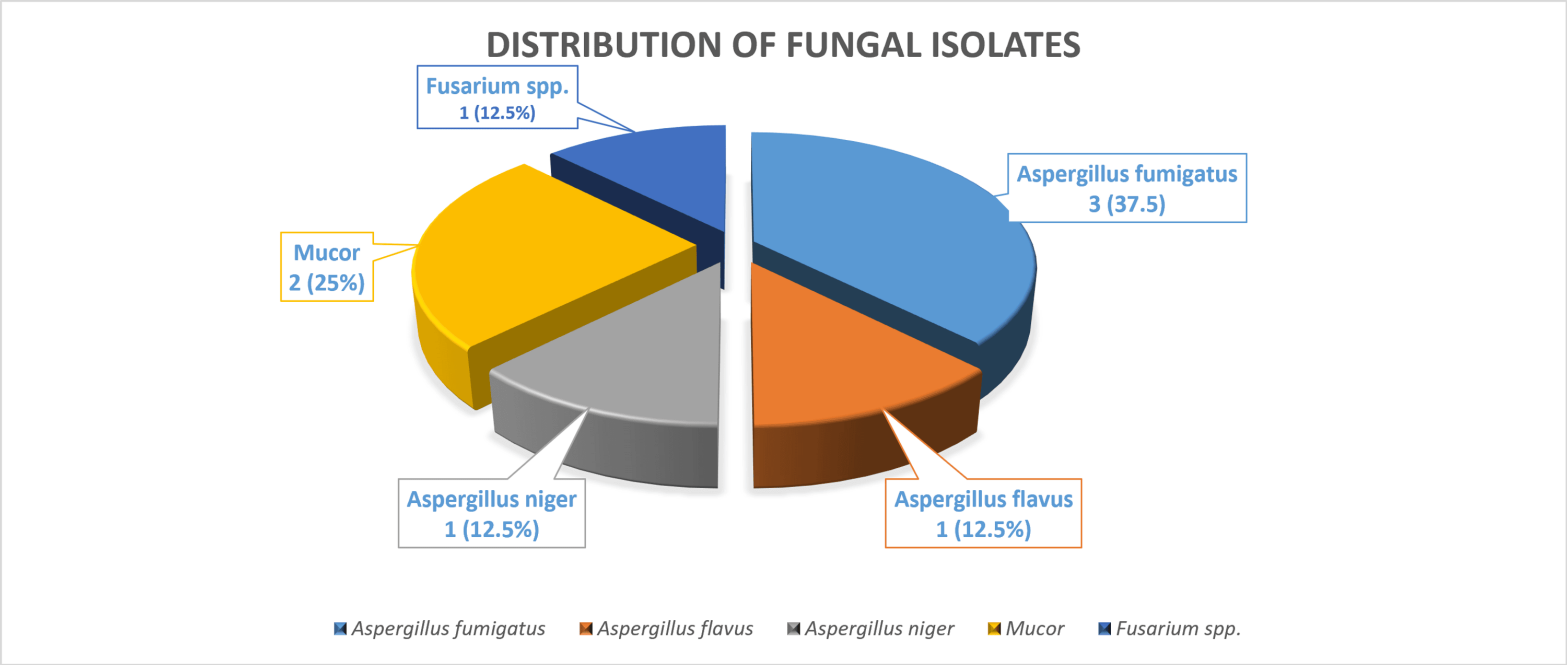
Distribution of fungal isolates in corneal ulcer cases. Data are presented as n, number; (%), percentage. No artificial intelligence (AI) tool was used to create the figure.

Figure 6 shows the distribution of fungal isolates recovered from culture-positive corneal ulcer cases. *A. fumigatus* was the predominant isolate, accounting for 3/8 (37.5%) cases, followed by *Mucor* spp. in 2/8 (25%) cases. *A. flavus*, *A. niger*, and *Fusarium* spp. were each isolated in 1/8 (12.5%) cases.

## Discussion

Fungal keratitis is recognized as a more chronic and severe corneal infection than bacterial keratitis and is associated with a higher incidence of corneal perforation and prolonged epithelial healing [11]. Fungal keratitis commonly follows ocular trauma, particularly with vegetative matter, which facilitates fungal invasion of the corneal stroma and progressive tissue damage if not diagnosed and treated promptly [12].

In the present study, fungal pathogens were isolated from 8 of 56 corneal ulcer samples (14.3%). *Aspergillus fumigatus* was the most frequently isolated species, accounting for 37.5% of culture-positive cases, followed by *Mucor *species (25%).

Most culture-positive cases occurred among farmers (62.5%), possibly reflecting increased exposure to agricultural environments, environmental fungal spores, and vegetative ocular trauma. However, the small number of culture-positive cases limits definitive conclusions regarding occupational risk.

As shown in Table 4, a male predominance was observed in the present study, which was consistent with the findings reported in previous Indian studies.

**Table 4:** Comparative study showing gender-wise distribution of patients Data are presented as percentages.

Authors	Year	Male%	Female%
Jisha et al^ [13]^	September 2011-August 2013	70.4	29.6
Vasudha et al^ [14]^	May 2018-July 2018	61	38.89
Present study	2022-2024	62.5	37.5

Similarly, as shown in Table 5, the majority of cases occurred in older age groups, in agreement with the observations of Kulkarni [15].

**Table 5:** Comparative study showing age-wise distribution of patients Data are presented as percentages.

Authors	Year	Age group affected in years (%)
Kulkarni^ [15]^	2020-2021	Above 60 (29.2)
Present study	2022-2024	41-60 and 61-80 each (37.5)

Furthermore, Table 6 demonstrates that *A. fumigatus* was the predominant fungal isolate in the present study, comparable to the findings reported by Waghmare and Sadanand and Sumangala and Sharlee [16,17].

**Table 6:** Comparative study showing distribution of fungal isolates in fungal keratitis Data are presented as percentages.

Authors	A. fumigatus	A. flavus	A. niger	Fusarium	Mucor
Waghmare and Sadanand^[16]^	33.33%	25%	0%	33.3%	0%
Sumangala and Sharlee^[17]^	37%	0%	0%	27%	0%
Present study	37.5%	12.5%	12.5%	12.5%	25%

Limitations

The findings of the present study should be interpreted in light of certain limitations. Only eight cases were culture-confirmed for fungal keratitis, which limits the generalizability of the observations. Furthermore, culture-based diagnosis may underestimate the true burden of fungal keratitis, as some clinically suspected cases may yield negative culture results despite infection. Therefore, the observed demographic and clinical characteristics should be considered descriptive findings rather than definitive epidemiological associations.

The sample size was relatively small (n = 56), which may limit the generalizability of the findings. As this was a single-center study, the results may not fully represent the broader epidemiological pattern of fungal keratitis in other regions.

In addition, the absence of a comparator group (such as bacterial or non-infective corneal ulcers) limits the ability to draw strong comparative conclusions regarding risk factors and causative organisms. Being an observational cross-sectional study, causal relationships between risk factors (such as ocular trauma or occupational exposure) and fungal infection could not be established.

Furthermore, only culture-positive fungal cases were analyzed in detail, which may introduce selection bias, and the relatively small number of positive isolates warrants cautious interpretation of proportions. Finally, fungal identification was performed using conventional methods without molecular confirmation, which may limit species-level precision.

## Conclusions

This study indicates that fungal pathogens were identified among corneal ulcer cases, with *A. fumigatus *being the most commonly isolated organism. The majority of fungal keratitis cases were observed in male patients with an agricultural background and a history of ocular trauma, particularly vegetative injury. Early microbiological diagnosis and prompt management are important to prevent vision-threatening complications in high-risk populations. However, the findings should be interpreted with caution due to the limited number of culture-positive cases and the absence of a comparator group.
